# Clinical and Radiological Characteristics of Cervical Spondylotic Myelopathy in Young Adults: A Retrospective Case Series of Patients under Age 30

**DOI:** 10.3390/medicina59030539

**Published:** 2023-03-10

**Authors:** Yoshiki Terashima, Takashi Yurube, Masatoshi Sumi, Aritetsu Kanemura, Koki Uno, Kenichiro Kakutani

**Affiliations:** 1 Department of Orthopaedic Surgery, Kobe Rosai Hospital, Kobe 651-0053, Japan; 2 Department of Orthopaedic Surgery, Kobe University Graduate School of Medicine, Kobe 650-0017, Japan; 3 Department of Orthopaedic Surgery, Mahoshi Hospital, Kobe 651-1242, Japan; 4 Department of Orthopaedic Surgery, Kobe Medical Center, Kobe 654-0155, Japan

**Keywords:** cervical spine, cervical spondylotic myelopathy (CSM), radiography, magnetic resonance imaging (MRI), sagittal spinal alignment, congenital spinal canal stenosis, dynamic spinal canal stenosis, intervertebral disc degeneration, spinal cord compression, young adult patients

## Abstract

*Background and Objectives:* Cervical spondylotic myelopathy (CSM) is a degenerative disease and occurs more frequently with age. In fact, the development of non-herniated CSM under age 30 is uncommon. Therefore, a retrospective case series was designed to clarify clinical and radiological characteristics of young adult patients with CSM under age 30. *Materials and Methods:* A total of seven patients, all men, with non-herniated, degenerative CSM under age 30 were retrieved from the medical records of 2598 hospitalized CSM patients (0.27%). Patient demographics and backgrounds were assessed. The sagittal alignment, congenital canal stenosis, dynamic canal stenosis, and vertebral slips in the cervical spine were radiographically evaluated. The presence of degenerative discs, intramedullary high-signal intensity lesions, and sagittal spinal cord compression on T2-weighted magnetic resonance images (MRIs) and axial spinal cord deformity on T1-weighted MRIs was identified. *Results:* All patients (100.0%) had relatively high daily sports activities and/or jobs requiring frequent neck extension. Cervical spine radiographs revealed the sagittal alignment as the “reverse-sigmoid” type in 57.1% of patients and “straight” type in 28.6%. All patients (100.0%) presented congenital cervical stenosis with the canal diameter ≤12 mm and/or Torg–Pavlov ratio <0.80. Furthermore, all patients (100.0%) developed dynamic stenosis with the canal diameter ≤12 mm and/or posterior vertebral slip ≥2 mm at the neurologically responsible segment in full-extension position. In MRI examination, all discs at the neurologically responsible level (100.0%) were degenerative. Intramedullary abnormal intensity lesions were detected in 85.7% of patients, which were all at the neurologically responsible disc level. *Conclusions:* Patients with non-herniated, degenerative CSM under age 30 are rare but more common in men with mild sagittal “reverse-sigmoid” or “straight” deformity and congenital canal stenosis. Relatively high daily activities, accumulating neck stress, can cause an early development of intervertebral disc degeneration and dynamic canal stenosis, leading to CSM in young adults.

## 1. Introduction

Cervical spondylotic myelopathy (CSM) is a neurological disorder induced by extrinsic compression of the spinal cord [1,2], resulting from developmental [3] and/or dynamic [4] canal stenosis with degenerative changes. Patients with CSM often complain of neck pain and also radicular pain, numbness, paresthesia, muscle weakness, and spasticity of the upper extremities [5]. Furthermore, CSM can introduce severe neurological damage including gait disturbance and bladder dysfunction, so that understanding the etiology and natural course of CSM is essential to provide the optimal treatment.

Cervical spondylotic myelopathy generally occurs as a result of degenerative changes affecting the vertebrae, intervertebral discs, facet joints, and associated ligaments in the spinal canal; therefore, the incidence of CSM increases with age [6]. While more than 50% of middle-aged patients show radiographic degeneration, 10% have clinically significant nerve root or spinal cord compression [6]. In our previous study of CSM, with the average age of 57.2 years, the youngest patient was 32 years old [7]. Thus, the development of CSM in young adults, especially under age 30, is very uncommon. To date, there have been several reports describing CSM in young adult patients [8,9], which however included patients with disc herniation as the study subject. Few studies exploring the pathomechanism of non-herniated, degenerative CSM in young adults have been published. The clarification of demographics, neurological symptoms, and radiological findings of young CSM patients would provide clinically useful information. Therefore, a retrospective case series was designed to elucidate clinical and radiological characteristics of CSM in young adult patients under age 30.

## 2. Materials and Methods

### 2.1. Study Design and Ethics Statement

The retrospective case series design was selected because of the focus on a disease with a low incidence. Data were collected from historical medical records at each facility under the approval and guidance of the Institutional Review Board (IRB) at Kobe University Graduate School of Medicine (No. B190002, 16 April 2019 approval). Considering the nature of the retrospective study design to review medical records of patients who completed the treatment, IRB waived the requirement to obtain informed consent. However, for the use of personal information and images of patients, although not identifiable, written informed consent for publication was obtained from each patient in accordance with the principles of the Declaration of Helsinki and the laws and regulations of Japan.

### 2.2. Patients

Patients were diagnosed with CSM based on clinical and neurological findings, radiographs, magnetic resonance images (MRIs), and/or myelographs. Patients who had disc herniation, flexion myelopathy, ossification of posterior longitudinal ligament, rheumatoid arthritis, athetoid-type cerebral palsy, or tumors were excluded. The diagnosis was obtained by accepting the majority decision of three senior spine clinicians blinded to the research purpose. We reviewed medical records of the three investigation sites, Kobe Rosai Hospital, Kobe Medical Center, and Kobe University Hospital (all located in Kobe, Japan), between 2001 and 2012, revealing that a total of 2598 CSM patients received hospitalization for surgical or intensive conservative treatment. Of the 2598 patients, only 7 patients were under age 30 (0.27%). All these patients underwent clinical follow-up for more than 2 years, who were therefore enrolled in this study.

### 2.3. Demographics, Backgrounds, and Neurological Characteristics

Patient demographics and backgrounds of sports activity, occupation, and trauma were obtained. Neurological assessment was performed by calculating the 1994 revised version of the Japanese Orthopaedic Association (JOA) scale score for cervical myelopathy (total 17 points) [10]. The segment responsible for compressive myelopathy was determined neurologically.

### 2.4. Radiography

**Figure 1:**
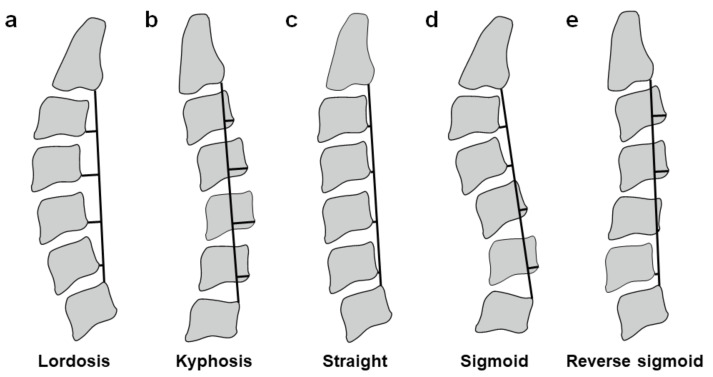
Schematic of the sagittal alignment of the cervical spine. On lateral cervical spine radiographs in neutral position, a line is drawn between the lower posterior edge of C2 and upper posterior edge of C7. The distances between the lower posterior edge of C3 to C6 and this line are measured. (**a**) When all the distances are anterior to the line and at least one is 2 mm or more, the sagittal alignment is defined as the “lordosis” type. (**b**) When all the distances are posterior to the line and at least one is 2 mm or more, the sagittal alignment is defined as the “kyphosis” type. (**c**) When all the distances are less than 2 mm anteriorly or posteriorly, the sagittal alignment is defined as the “straight” type. (**d**) When the upper cervical distance(s) is anterior but the lower cervical distance(s) is posterior, the sagittal alignment is defined as the “sigmoid” type. (**e**) When the upper cervical distance(s) is posterior but the lower cervical distance(s) is anterior, the sagittal alignment is defined as the “reverse sigmoid” type.

Lateral cervical spine radiographs were taken in neutral, full-flexion, and full-extension positions under a standardized protocol (exposure time 80 ms; distance 150 cm; current 250 mA; voltage 72 kV) with the use of a scale marker [11,12,13,14]. First, to understand the cervical spine sagittal alignment, we classified the sagittal alignment in neutral position into the five types of “lordosis”, “kyphosis”, “straight”, “sigmoid”, and “reverse sigmoid”, according to the classification reported by Matsumoto M et al. [15] (Figure 1). Then, to recognize congenital cervical stenosis, we measured the anteroposterior spinal canal diameter from the midpoint of the posterior surface of the vertebral body to the closest point of the spinolaminar line, which is called the “developmental factor (DVF)” [3]. The DVF distance of 12 mm or less was defined as positive [3]. As an alternative method, we calculated the “Torg–Pavlov ratio” by dividing the sagittal spinal canal diameter by the anteroposterior vertebral body diameter at the same level [16]. The ratio of less than 0.8 was defined as positive [17]. Next, to identify dynamic cervical stenosis, we measured the distance between the lower posterior corner of the vertebral body and the upper base of the spinous process of the lower adjacent vertebra in full-extension position, which is called the “dynamic factor (DNF)” [4]. The DNF distance of 12 mm or less was defined as positive [4]. We additionally assessed the presence of vertebral slips. We defined the anterior or posterior translation of 2 mm or more as positive in full-flexion or full-extension position [11,12,13,14]. Radiographs were assessed twice at a one-week interval by each of two spine surgeon examiners who were blinded to this study. In the sagittal alignment of the cervical spine, the majority decision of the total four-time measurements was accepted. In radiographic parameters, the average values of these measurements were used for evaluation.

### 2.5. Magnetic Resonance Imaging

Routine MRIs of the cervical spine were taken using a 1.5-T system with the standardized protocol (axial T1-weighted spin echo imaging: repetition time (TR) 600 ms, echo time (TE) 20 ms, slice thickness (ST) 5.0 mm, slice gap (SG) 0 mm, field of view (FOV) 220 mm; sagittal T2-weighted turbo spin echo (TSE) imaging: TR 3000 ms, TE 120 ms, ST 0.4 mm, SG 0.4 mm, TSE factor 15, FOV 300 mm; axial T2-weighted TSE imaging: TR 2500 ms, TE 90 ms, ST 5.0 mm, SG 0 mm, TSE factor 13, FOV 220 mm) [7]. We classified the severity of intervertebral disc degeneration according to the Pfirrmann classification [18]. Discs with the grades III–V were defined as “degenerative”. We also assessed the severity of spinal cord compression on T2-weighted sagittal images by the Takahashi classification [19] and defined the compressed spinal cord with the degrees 2–3 as positive. Similarly, we also evaluated the presence of intramedullary increased signal intensity changes on T2-weighted images as a reference to determine the neurologically responsible segment. The abnormal signal was defined as a high-intensity area in contrast to adjacent isointense portions of the spinal cord [9,20,21,22], which were graded according to the system described by Mehalic TF et al. [21]. Lesions with the grades 2–4 were defined as positive. In addition, the severity of spinal cord deformity on T1-weighted axial images was analyzed and categorized into the two types: “ovoid” and “angular-edged” [7]. The “angular-edged” deformity was defined as a poor prognostic factor [7]. Magnetic resonance images were analyzed twice at a one-week interval by two radiologists blinded to this study individually, and the presence of lesions was determined by the majority decision of the total four-time measurements.

### 2.6. Statistical Analysis

Data are expressed as the mean ± standard deviation. In radiographic and MRI measurements, the intra-class correlation coefficient and κ coefficient were calculated to assess the intra-observer and inter-observer reliability for parametric and non-parametric variables, respectively.

## 3. Results

### 3.1. Clinical Characteristics

Patient demographics, backgrounds, and neurological findings are summarized in Table 1. All patients were men with the mean age of 23.3 ± 3.7 years (range: 17–28). Their mean JOA score at the initial visit was 12.5 ± 2.4 (range: 7.5–16). Neurologically responsible level was C3–C4 in three patients, C5–C6 in three patients, and C4–C5 in one patient. They had relatively high daily sports activities, accumulating neck stress, which consisted of two Judo, three football, and one baseball players (total six of seven patients, 85.7%). A patient without any sports experience worked as a pastry chef requiring frequent neck extension during laboring. In addition, two of seven patients (28.6%) had episodes of neck injury by a traffic accident.

### 3.2. Radiographic Characteristics

Radiographic findings are summarized in Table 2. The intra-observer reliability by the intra-class correlation coefficient for parametric Torg–Pavlov ratio, DVF, DNF, and vertebral slip was 0.97–0.98, 0.88–0.92, 0.85–0.91, and 0.93–0.97, respectively. The inter-observer reliability by the intraclass correlation coefficient was 0.97, 0.93, 0.86, and 0.93, respectively. The intra-observer and inter-observer reliability by κ coefficient for non-parametric sagittal alignment of the cervical spine was 0.77–0.78 and 0.78, respectively. All the values indicated a satisfactory reproducibility.

In the cervical spine sagittal alignment, the “reverse-sigmoid” type was most frequently seen in four of seven patients (57.1%). Of these patients, three with the “reverse-sigmoid” type (42.9%) had the single-level spinal cord compression in MRI examination, which was all found at the transitional segment between kyphotic and lordotic levels with the positive DNF and/or posterior vertebral slip. Although a patient with the “reverse-sigmoid” type had the multi-level spinal cord compression, T2-weighted signal hyperintensity at the transitional segment was also detected in MRI. Meanwhile, in the other three of seven patients (42.9%), one (14.3%) displayed the “lordosis” type and two (28.6%) exhibited the “straight” type. Then, all these patients (42.9%) had the multi-level spinal cord compression in MRI. Then, congenitally narrowed cervical spinal canal was detected in four of seven patients (57.1%) based on DVF ≤12 mm and in all patients (100.0%) based on the Torg–Pavlov ratio <0.80. In addition, six of seven patients (85.7%) presented the positive DNF. Positive vertebral slips were also observed in four of seven patients (57.1%), which were all the posterior translation in full-extension position. Furthermore, in all patients, the neurologically responsible segment was matched with levels of the positive DNF and/or posterior vertebral slip.

### 3.3. Magnetic Resonance Imaging Characteristics

The findings of MRI are summarized in Table 3. The intra-observer reliability by the κ coefficient for non-parametric intervertebral disc degeneration, sagittal spinal cord compression, intramedullary abnormal signal intensity, and axial spinal cord deformity was 0.80–0.84, 0.88–0.94, 1.00, and 1.00, respectively. The inter-observer reliability by the κ coefficient was 0.80, 0.94, 1.00, and 1.00, respectively. Outcomes of reliability analysis were all acceptable.

Intervertebral discs at the neurologically responsible segment were all “degenerative”, which was identified as the Pfirrmann grades III–IV. Intramedullary increased signal intensity changes on T2-weighted images were positive in six of seven patients (85.7%). Notably, the location of these intramedullary abnormal signal changes was all matched with the responsible segment assessed by neurological examination. Although a patient case had two abnormal signal intensity lesions of the spinal cord at C3–C4 and C4–C5 levels, we determined the responsible segment as C3–C4 based on his neurological findings. Then, six of seven patients (85.7%) had the positive spinal cord compression. Furthermore, four of seven patients (57.1%) had the “angular-edged” deformity at the neurologically responsible level.

### 3.4. Case Reports

**Figure 2:**
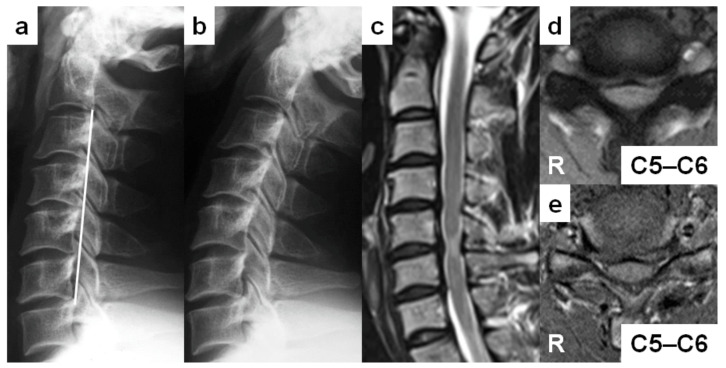
Report of a 27-year-old man with cervical spondylotic myelopathy (case no. 1). (**a**,**b**) Lateral cervical spine radiographs in neutral (**a**) and full-extension (**b**) positions demonstrating the “straight” cervical sagittal alignment. (**c**) A sagittal T2-weighted magnetic resonance image (MRI) demonstrating “degenerative” discs from C3–C4 to C6–C7, spinal cord compression from C4–C5 to C6–C7, and an intramedullary increased signal intensity lesion at C5–C6. (**d**) An axial T2-weighted MRI demonstrating the narrowed cervical spinal canal with an intramedullary increased signal intensity area at C5–C6. (**e**) An axial T1-weighted MRI demonstrating the “angular-edged” spinal cord deformity at C5–C6.

A 27-year-old man (case no. 1) was referred to our outpatient clinic with complaints of numbness in both ring and little fingers lasting several months. He was a postman and played Judo. He had modest muscle weakness on the left fingers. The Romberg sign was positive. The JOA score at the initial visit was 14 of 17 points. On lateral cervical spine radiographs, the cervical sagittal alignment was “straight” (Figure 2a). The Torg–Pavlov ratio was below 0.80 from C3 to C7. The DVF was positive from C4 to C7. The DNF was also positive at C5–C6, although no obvious vertebral slip was found in full-extension position (Figure 2b). On T2-weighted sagittal MRIs, “degenerative” discs existed from C3–C4 to C6–C7 (Figure 2c). Multi-level spinal cord compression was detected at C4–C5 to C6–C7. An intramedullary increased signal intensity lesion was confirmed at C5–C6, corresponding with the neurologically responsible segment (Figure 2c,d). On T1-weighted axial MRIs, the “angular-edged” spinal cord deformity was observed at C5–C6 (Figure 2e). He took surgery of open-door laminoplasty. His neurological symptoms disappeared with a fully marked JOA score (17 of 17 points) two years after surgery.

**Figure 3:**
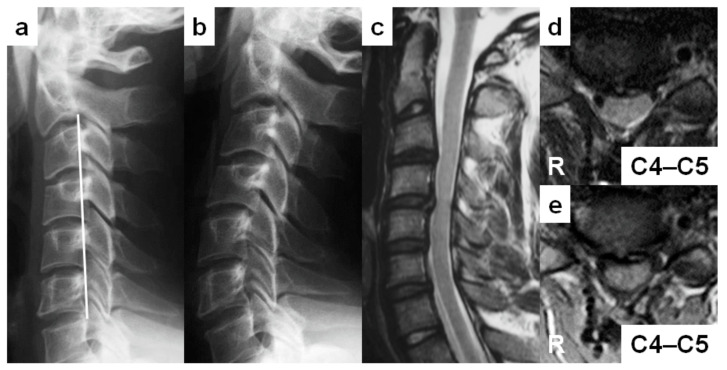
Report of a 24-year-old man with cervical spondylotic myelopathy (case no. 3). (**a**,**b**) Lateral cervical spine radiographs in neutral (**a**) and full-extension (**b**) positions demonstrating the “reverse-sigmoid” sagittal cervical alignment and posterior vertebral slip at C4. (**c**) A sagittal T2-weighted magnetic resonance image (MRI) demonstrating “degenerative” discs from C3–C4 to C6–C7, spinal cord compression at C4–C5, and no intramedullary increased signal intensity lesions. (**d**) An axial T2-weighted MRI demonstrating the narrowed cervical spinal canal without any intramedullary increased signal intensity areas at C4–C5. (**e**) An axial T1-weighted MRI demonstrating the “ovoid” spinal deformity at C4–C5.

A 24-year-old man (case no. 3)’s complaint was right finger numbness when the neck was extended. He had no sports activities but was a pastry chef requiring frequent neck extension. He demonstrated the negative Romberg sign, normal deep tendon reflexes, and modest sensory disturbance on his right finger with 16 of 17 points in the JOA score. His cervical sagittal alignment was “reverse sigmoid” (Figure 3a). The Torg–Pavlov ratio was positive from C3 to C7, although the DVF was negative throughout. Positive DNF at C4–C5 level and posterior vertebral slip at C4 were found in full-extension position (Figure 3b). “Degenerative” discs existed from C3–C4 to C6–C7 (Figure 3c). There was no signal hyperintensity on T2-weighted MRIs, but the positive spinal cord compression was seen at C4–C5 (Figure 3c,d). On T1-weighted MRIs, “ovoid” spinal cord deformity was shown at the neurologically responsible segment of C4–C5 (Figure 3e). No foraminal stenosis or peripheral nerve disorder were detected. After C4–C5 anterior cervical discectomy and fusion, his neurological condition had a full recovery one year after surgery.

## 4. Discussion

In the present study, only 7 of 2598 CSM patients (0.27%) who received inpatient hospital care between 2001 and 2012 were under age 30. They were all men and had relatively high daily sports activities and/or jobs requiring frequent neck extension. Radiographs revealed a markedly high prevalence of congenitally narrowed cervical spinal canals, with the positive DVF and/or Torg–Pavlov ratio, and dynamic spinal movements, with the positive DNF and/or vertebral slippage. Then, MRIs clarified a frequent involvement of intervertebral disc degeneration, spinal cord compression, and intramedullary abnormal signal changes at the neurologically responsible level. In the cervical spine sagittal alignment, the “reverse-sigmoid” type was the most common and often developed the single-level spinal cord compression at the transitional segment between kyphotic and lordotic levels, whereas the “straight” type was associated with the multi-level spinal cord compression but with the single-level dynamic canal stenosis and spinal cord damage at the neurologically responsible segment. These findings suggest that high daily activities, requesting neck stress, would facilitate an early development of disc degeneration, dynamic canal stenosis, and spinal cord compression and damage, ultimately leading to symptomatic CSM in young adult patients with mild sagittal malalignment including the “reverse-sigmoid” or “straight” type and congenital canal stenosis.

Sports activities are an important cause of spinal injuries among young people. Sports-related spinal injuries accounted for 11% of all acute spinal injury admissions [23]. Contact sports such as American football and rugby football have a high risk of spinal injuries [24]. Severe judo-related neck injuries have also been reported [25]. In the present study, all patients demonstrated relatively high daily activities of sports and/or occupations, indicating neck stress. While many reported cases showed high-energy, sudden onsets during the activities [23,24,25], our patients had no episodes about the immediate development of symptoms. Therefore, the accumulation of daily, mild injuries and traumas could enhance degenerative spinal changes and/or subclinical neurological damage.

Congenitally narrowed cervical spinal canal stenosis is a known risk factor for CSM [3,26]. Measuring the sagittal spinal canal diameter on lateral radiographs is the most common method [3]. Risks of CSM development would appear to be high when the sagittal spinal canal diameter is 12 mm or less [3]. Then, to reduce the variation in the radiographic magnification, the ratio calculation has been developed [16]. The Torg–Pavlov ratio has significantly been smaller in myelopathic patients than in non-spondylotic, non-myelopathic patients, which could reflect the likelihood of developing CSM [27]. In this study, although the positive DVF was identified in four of seven patients (57.1%), all patients displayed the Torg–Pavlov ratio <0.8, indicating the predisposition of the congenitally narrowed canal space. In general, the prevalence of congenitally narrowed cervical spinal canal stenosis is higher in women than in men [28]. Therefore, high daily activities, often experienced in men, could become a relatively robust contributor to spinal cord compression in subjects with congenital canal stenosis.

Another risk factor for CSM is dynamic canal stenosis. The concept has been proposed as the “pincer mechanism” [29]. On lateral radiographs, the canal diameter of 12 mm or less in full-extension position is a predictor to develop CSM [4]. In the current study, DNF was positive in six of seven patients (85.7%). In addition, a patient with the negative DNF had the positive posterior vertebral slip at the neurologically responsible segment. Therefore, the comorbidity of dynamic canal stenosis, possibly occurring with high daily activities, in people with congenital canal stenosis could accelerate spinal cord compression.

The assessment of cervical spine instability and canal stenosis based on radiographic measurements of absolute values is controversial, although we used a standardized exposure protocol with placing a scale marker to reduce the varying magnification. However, at the C1 level, prior studies have suggested the two criteria in the space available for the spinal cord for myelopathy of 13 mm or less [30,31] and 14 mm or less [32]. Then, a subsequent receiver operating characteristic curve analysis has identified the adequacy of both the two cut-off points [33]. These pieces of evidence support diagnostic values of cervical spine parameter measurements using absolute numbers, which are still the most commonly used in the clinical situation. In patients who fulfill the radiographic criteria for instability and stenosis, further evaluation with MRI for the multidisciplinary diagnosis of myelopathy is warranted.

Magnetic resonance imaging can provide the direct clinical evidence regarding intervertebral disc and spinal cord pathology. In this study, all discs (100.0%) at the neurologically responsible level were “degenerative” with the Pfirrmann grades III–IV. The Pfirrmann classification of intervertebral disc degeneration, originally developed for the lumbar spine, has become well applicable for the cervical spine [34]. According to the Takahashi classification, six of seven patients (85.7%) showed the positive sagittal spinal cord compression. Then, four of seven (57.1%) demonstrated the “angular-edged” axial spinal cord deformity. Furthermore, intramedullary abnormal signal lesions, a common poor prognostic factor of CSM [35], were also frequently detected at the neurologically responsible disc level. Taken together, in patients with congenitally narrowed canals, (1) high daily activities could induce early disc degeneration, (2) degenerative instability could cause dynamic canal stenosis, (3) the combination of congenital and dynamic canal stenosis could further promote spinal cord compression and then damage, and (4) the chronic or traumatic accumulation of spinal cord damage could possibly reach the development of symptomatic CSM.

More specifically, to find radiographic and biomechanical characteristics of CSM in young adult patients, we assessed the cervical spine sagittal alignment. The C2–C7 Cobb angle is often used to evaluate the sagittal alignment, which however gives only an indication of the ends of the curve, resulting in the underestimation of the local lordosis and kyphosis [36]. In this viewpoint, we employed the Matsumoto classification [15]. Consequently, the “reverse-sigmoid” sagittal alignment was the most frequently observed. Many patients with the “reverse-sigmoid” type had the MRI-based single-level spinal cord compression at the transitional segment between kyphotic and lordotic levels with the positive DNF and/or posterior vertebral slip. A patient with the MRI-based multi-level spinal cord compression also had the positive DNF and spinal cord compression at the transitional segment. This “reverse-sigmoid” alignment could induce mechanical overload to the transitional disc. The other patients with “lordosis” or “straight” alignment had the multi-level spinal cord compression in MRI evaluation; however, these patients also developed the single-level positive DNF or posterior vertebral slippage all with the intramedullary abnormal signal intensity at the neurologically responsible level, suggesting the altered biomechanics. Therefore, young male athletes and/or workers with baseline sagittal “reverse-sigmoid” or “straight” malalignment and congenital canal stenosis should have careful clinical follow-up to identify dynamic canal stenosis and spinal cord damage at the neurologically responsible segment.

Caution in interpreting our results is advised. First, the actual prevalence of CSM under age 30, including patients with mild symptoms treated in outpatient clinics, still remains unclear, due to the inclusion of hospitalized patients only. Second, the small study sample size of only seven patients indicates low statistical power, causing possible interpretation errors, despite the appropriate statistical analysis methods. Third, this study does not have the control group. We tried to analyze 2591 of 2598 patients with CSM over aged 30, but too big a difference in sample size was expected to statistically preclude conclusion. Finally, the retrospective design of this study provides only limited evidence because of the difficulty to exclude the selection bias; however, the observed distinct characteristics of non-herniated, degenerative CSM in young adult patients raise the necessity of future prospective cohort studies.

## 5. Conclusions

Young adult patients with non-herniated, degenerative CSM under aged 30 are rare, but they are more common in men who have backgrounds of congenital canal stenosis with mild sagittal “reverse-sigmoid” or “straight” deformity. In these subjects, high daily activities of sports and/or jobs, building up neck stress, would facilitate an early development of intervertebral disc degeneration and dynamic canal stenosis at the biomechanically transitional segment in the cervical spine, leading to spinal cord compression, damage, and possibly symptomatic myelopathy.

## Figures and Tables

**Table 1:** Clinical characteristics of young adult patients with cervical spondylotic myelopathy under age 30.

Case No.	Age (Years)	Sex	Duration of Symptoms (Years)	Sports Activity	Occupation	Trauma	Neurologically Responsible Disc Level	Baseline JOA Score (/17 Points)	Surgery	Endpoint JOA Score (/17 Points)
1	27	Man	0.5	Judo	Postman	−	C5–C6	14	+	17
2	28	Man	0.5	Judo	Police officer	−	C5–C6	13	−	13
3	24	Man	0.1	−	Pastry chef	Traffic accident	C4–C5	16	+	17
4	23	Man	2	Football	−	Traffic accident	C5–C6	13	+	16
5	19	Man	0.3	Baseball	College student	−	C3–C4	7.5	+	13
6	17	Man	1	Football	Sushi chef	−	C3–C4	11	+	14
7	25	Man	1	Football	Gas station attendant	−	C3–C4	13	+	16

JOA, Japanese Orthopedic Association.

**Table 2:** Radiographic characteristics of young adult patients with cervical spondylotic myelopathy under age 30.

Case No.	Sagittal Alignment	Torg–Pavlov Ratio		DVF		DNF		Vertebral Slip			
		<0.8	Level	≤12 mm	Level	≤12 mm	Level	≥2 mm	Level	Direction	Position
1	Straight	+	C3, C4, C5, C6, C7	+	C4, C5, C6, C7	+	C5–C6	−			
2	Straight	+	C3, C4, C5, C6, C7	+	C4, C5, C6	+	C5–C6	−			
3	Reverse sigmoid	+	C3, C4, C5, C6, C7	−		+	C4–C5	+	C4	Posterior	Extension
4	Reverse sigmoid	+	C3, C4, C5, C6, C7	+	C4, C5, C6	+	C5–C6	−			
5	Reverse sigmoid	+	C3, C4, C5, C6, C7	+	C4, C5, C6	+	C3–C4, C4–C5, C5–C6	+	C3	Posterior	Extension
6	Reverse sigmoid	+	C3, C4, C5, C6, C7	−		+	C3–C4	+	C3	Posterior	Extension
7	Lordosis	+	C3, C4, C5, C6	−		−		+	C3	Posterior	Extension

DVF, developmental factor; DNF, dynamic factor.

**Table 3:** Magnetic resonance imaging characteristics of young adult patients with cervical spondylotic myelopathy under age 30.

Case No.	Intervertebral Disc Degeneration		Sagittal Spinal Cord Compression		Intramedullary Abnormal Signal Intensity		Axial Spinal Cord Deformity at the Neurologically Responsible Level
	Positive	Level	Positive	Level	Positive	Level	
1	+	C3–C4, C4–C5, C5–C6, C6–C7	+	C4–C5, C5–C6, C6–C7	+	C5–C6	Angular edged
2	+	C2–C3, C3–C4, C4–C5, C5–C6, C6–C7	+	C4–C5, C5–C6, C6–C7	+	C5–C6	Angular edged
3	+	C3–C4, C4–C5, C5–C6, C6–C7	+	C4–C5	−		Ovoid
4	+	C2–C3, C3–C4, C4–C5, C5–C6, C6–C7	+	C5–C6	+	C5–C6	Ovoid
5	+	C3–C4, C4–C5, C5–C6	+	C3–C4, C4–C5, C5–C6	+	C3–C4, C4–C5	Angular edged
6	+	C2–C3, C3–C4, C4–C5	−		+	C3–C4	Angular edged
7	+	C2–C3, C3–C4, C4–C5, C5–C6	+	C3–C4, C4–C5, C5–C6	+	C3–C4	Ovoid

## Data Availability

The data presented in this study are available on request from the corresponding author.
